# Age at menopause, *APOE-ε4*, and Alzheimer’s disease risk

**DOI:** 10.21203/rs.3.rs-10121551/v1

**Published:** 2026-07-22

**Authors:** Madeline Wood Alexander, Alexander J. Nyman, Kaitlin B. Casaletto, Rowan Saloner, Katie L. Vandeloo, Tallinn F. L. Splinter, Walter Swardfager, Julie Ottoy, Mario Masellis, Liisa A. M. Galea, Sandra E. Black, Gillian Einstein, Marisa N. Denkinger, Nicholas J. Ashton, Jessica Z. K. Caldwell, Sterling C. Johnson, Zoe Arvanitakis, Jennifer S. Rabin

**Affiliations:** 1 Hurvitz Brain Sciences Program, Sunnybrook Research Institute, Toronto, Ontario, Canada; 2 Rehabilitation Sciences Institute, Temerty Faculty of Medicine, University of Toronto, Ontario, Canada; 3 Fein Memory and Aging Center, Department of Neurology, Weill Institute for Neurosciences, University of California, San Francisco, CA, USA; 4 Ann S. Bowers Women’s Brain Health Initiative, University of California, California, USA; 5 Department of Psychological Clinical Sciences, University of Toronto Scarborough, Ontario, Canada; 6 Department of Pharmacology & Toxicology, University of Toronto, Ontario, Canada; 7 Centre for Brain Resilience and Recovery, Hurvitz Brain Sciences Program, Sunnybrook Research Institute Toronto, Ontario, Canada; 8 Division of Neurology, Department of Medicine, Sunnybrook Health Sciences Centre, University of Toronto, Toronto, Ontario, Canada; 9 Campbell Family Mental Health Research Institute, The Centre for Addition and Mental Health, Toronto, Ontario, Canada; 10 Department of Psychiatry, Temerty Faculty of Medicine, University of Toronto, Toronto, Ontario, Canada; 11 Rotman Research Institute, Baycrest Hospital, University of Toronto, Toronto, Ontario, Canada; 12 Dalla Lana School of Public Health, University of Toronto, Toronto, Ontario, Canada; 13 Department of Psychology, University of Toronto, Ontario, Canada; 14 Banner Sun Health Research Institute, Sun City, AZ, USA; 15 Banner Alzheimer’s Institute, Phoenix, AZ, USA; 16 Wisconsin Alzheimer’s Disease Research Center, School of Medicine and Public Health, University of Wisconsin, Madison, WI, USA; 17 Wisconsin Alzheimer’s Institute, University of Wisconsin, Madison, WI, USA; 18 Cleveland Clinic Lou Ruvo Center for Brain Health, Las Vegas, NV, USA; 19 Rush Alzheimer’s Disease Center, Rush University Medical Center, Chicago, IL, USA; 20 Harquail Centre for Neuromodulation, Sunnybrook Health Sciences Centre, University of Toronto, Toronto, Ontario, Canada

## Abstract

**Importance:**

*APOE-ε4* is an established risk factor for Alzheimer’s disease (AD) and confers greater risk in women than in men. Earlier age at menopause also increases AD risk in women. Yet whether menopause timing influences *APOE-ε4*-related AD risk remains unclear.

**Objective:**

To examine whether age at menopause modifies the association of *APOE-ε4* with AD risk.

**Design, setting and participants:**

Data were analyzed from postmenopausal women free from known dementia at study entry in two longitudinal datasets: (1) the harmonized data from the Religious Orders Study, Rush Memory and Aging Project, and Minority Aging Research Study (ROS/MAP/MARS), and (2) the Wisconsin Registry for Alzheimer’s Prevention (WRAP). Data were collected between 1994–2025.

**Main outcomes and measures:**

In both datasets, neuropsychological tests assessed longitudinal memory performance, and MRI quantified cortical thickness and brain volume in AD vulnerable regions. In WRAP, which included *in vivo* AD biomarkers, AD pathology was assessed using longitudinal plasma p-tau217 and cross-sectional beta-amyloid (Aβ) PET. Menopause history was self-reported, and *APOE* status was classified as *ε4* carrier vs. non-carrier. Linear mixed-effects or linear regression models were used, as appropriate, to test interactions between *APOE-ε4* carrier status and age at menopause on memory decline, brain atrophy, p-tau217 accumulation, and Aβ-PET burden, adjusting for relevant covariates.

**Results:**

The study included 2,625 women in ROS/MAP/MARS (mean [*SD*] age=77.4 [7.77], mean [SD] age at menopause=47.9 [7.10]) and 512 women in WRAP (mean [SD] age=60.2 [5.57], mean [SD] age at menopause=50.1 [6.29]). In both datasets, earlier age at menopause strengthened associations of *APOE-ε4* with memory decline (ROS/MAP/MARS: β=0.068, *p*=.02; WRAP: β=0.092, *p*=.03) and MRI measures of brain atrophy (ROS/MAP/MARS: β=0.071, *p*=.05; WRAP: β=0.256, *p*=.03). In WRAP, earlier menopause also amplified associations of *APOE-ε4* with p-tau217 accumulation (β=−0.029, *p*=.05) and global Aβ-PET burden (β=−0.146, *p*=.01).

**Conclusions and relevance:**

Earlier menopause strengthened the associations between *APOE*-ε4 and key AD outcomes. These findings suggest that menopause timing may influence *APOE*-ε4-related susceptibility to AD, highlighting midlife endocrine processes as potential targets for prevention in women.

## 1. Introduction

Apolipoprotein E (*APOE*) is the strongest genetic risk factor for late-onset Alzheimer’s disease (AD).^1–5^ The *ε4* allele accelerates key AD processes, including beta-amyloid (Aβ) and tau accumulation, brain atrophy, and cognitive decline.^1–5^ Importantly, *APOE-ε4* exhibits well-established sex-specific effects, conferring greater risk for AD in women than in men.^6–12^ These differences likely contribute to the nearly twofold higher lifetime risk of AD in women.^13,14^

Another factor that may contribute to women’s elevated risk for AD is menopause. The menopause transition is characterized by a sharp decline in 17β-estradiol and a concurrent rise in follicle-stimulating hormone (FSH), a neuroendocrine shift that has been shown to promote AD pathogenesis in animal models.^15,16^ Although menopause itself may accelerate age-related neurodegenerative processes,^17^ earlier age at menopause is associated with even greater risk.^18–21^ The mechanisms linking menopause timing to AD are unclear, but growing evidence suggests that earlier menopause is associated with increased vulnerability to several dementia risk processes, including worse vascular health, synaptic loss, and both AD and non-AD neuropathologies.^18,22–24^

Apolipoprotein E and female-specific endocrine processes may converge through shared biological pathways in the brain, including regulation of immune responses, vascular function, and synaptic plasticity.^25–28^ Accordingly, *APOE* genotype may modify the effects of menopause on AD risk. Supporting this possibility, preclinical studies show that *APOE-ε4* carriers exhibit greater accumulation of AD pathology and more pronounced cognitive deficits in response to the endocrine changes associated with menopause.^15,29^ Limited evidence in humans suggests a similar interaction may exist. Specifically, *APOE*-ε4 amplifies associations of bilateral oophorectomy (i.e., surgical menopause) with AD risk and strengthens associations between earlier age at menopause and worse cognition later in life.^30,31^ Some studies also suggest that *APOEε4* modifies associations between menopausal hormone therapy and AD outcomes, although the direction of these effects remain inconsistent.^32,33^

Despite the key roles of both *APOE-ε4* and menopause timing in women’s risk for AD, whether these factors interact to influence AD outcomes has not been systematically examined. In the present study, we investigated whether age at menopause modifies associations between *APOE-ε4* and AD endophenotypes in two independent datasets of postmenopausal women. We hypothesized that women with earlier menopause would exhibit greater susceptibility to the detrimental effects of *APOE-ε4* on memory decline, brain atrophy, and *in vivo* AD biomarkers.

## 2. Methods

### 2.1. Participants

We used data from two sources: (1) three Rush Alzheimer’s Disease Centre cohort studies (i.e., the Religious Orders Study (ROS),^34^ the Rush Memory and Aging Project (MAP),^34^ and the Minority Aging Research Study (MARS)^35^), and (2) the Wisconsin Registry for Alzheimer’s Prevention (WRAP).^36^ ROS/MAP/MARS and WRAP are longitudinal observational studies of aging. WRAP is enriched with participants with a parental history of AD, while ROS/MAP/MARS comprises community-dwelling older adults. In both datasets, we used data from study inception through November 2025. All studies received institutional ethics approval, and all participants provided written informed consent. We followed the Strengthening the Reporting of Observational Studies in Epidemiology guidelines for cohort studies.

In both datasets, participants self-reported their sex as “female” or “male,” with no data collected on sex at birth vs. gender identity. We refer to female participants as “women” and male participants as “men,” acknowledging that sex and gender are conflated.^37^ Primary analyses included postmenopausal women who had available data on age at menopause, *APOE* genotype, main covariates, and at least one cognitive or brain outcome examined (eFigure1). To contextualize associations observed in women, we also examined associations of *APOE-ε4* status and AD endophenotypes in male participants with available data on *APOE* genotype, covariates, and study endophenotypes.

### 2.2. *APOE* status

The single-nucleotide polymorphisms rs429358 and rs7412 were used to classify *APOE* genotype. In primary analyses, we compared *APOE-ε4* carriers (i.e., *ε3/ε4* or *ε4/ε4*) to non-carriers (i.e., *ε3/ε3, ε2/ε3* or *ε2/ε2*). *APOE ε2/ε4* carriers were excluded because *ε2* and *ε4* alleles exert opposite effects on AD risk.^38^ Both datasets met Hardy-Weinberg Equilibrium expectations.^39^

### 2.3. Menopause

In ROS/MAP/MARS, women self-reported the age at which they stopped menstruating and whether menopause occurred naturally (i.e., spontaneously) or by surgery. In WRAP, postmenopausal women reported their age at last menstrual period, and if applicable, histories and ages of hysterectomy or oophorectomy. Following clinical conventions,^40^ age at menopause was defined as one year after the last menstrual period for women with spontaneous menopause and as age at the last menstrual period for women with surgical menopause. In WRAP, which captured hysterectomy and oophorectomy status, we excluded women with hysterectomy without oophorectomy (*N*=22), as menstrual cessation may not reflect ovarian cessation when the ovaries are intact. We also excluded women who reported highly improbable ages at menopause (last menstrual period at <20 or ≥70 years; *N*=15 in ROS/MAP/MARS; *N*=2 in WRAP). Women also self-reported their history of menopause hormone therapy use.

### 2.4. Cognition

Cognition was assessed using standardized neuropsychological tests administered approximately annually in ROS/MAP/MARS and approximately every two years in WRAP.^34,36^ We focused on composite scores of episodic memory which typically shows the earliest decline in AD.^41^ In ROS/MAP/MARS, the score included standardized scores on a Word List recall and recognition measure,^42^ East Boston Story immediate and delayed recall,^43^ and Logical Memory immediate and delayed recall.^44^ In WRAP, the score included standardized immediate and delayed recall scores on the Rey Auditory-Verbal Learning Test (RAVLT)^45^, Logical Memory,^44^ and the Brief Visuospatial Memory Test–Revised (BVMT-R).^46^

In both datasets, cognitive status is assessed at each visit, and diagnoses of mild cognitive impairment (MCI) and dementia are made through consensus processes that consider neuropsychological test scores and corroborating clinical findings.^36,47^

### 2.5. Brain Magnetic Resonance Imaging (MRI)

In both datasets, subsets of participants underwent 3T brain MRI including a T1-weighted structural scan. In ROS/MAP/MARS, participants were scanned on either a UC 3T Philips Achieva, a Morton Grove 3T Siemens Trio, or a RIRC 3T Siemens Prisma, using 32 channel coils. T1w images were acquired using 3D magnetizationprepared rapid acquisition gradient-echo (MPRAGE) sequences.^48^ Then, distortion-corrected MPRAGE images were segmented into Desikan-Killiany cortical and subcortical regions of interest (ROIs) using FreeSurfer (surfer.nmr.mgh.harvard.edu). Regional cortical thickness values were generated by averaging the thickness of homologous ROIs in contralateral hemispheres. In WRAP, participants were scanned on a GE 3T MR750 (Waukesha, WI, USA) with an 8 channel coil, and T1w images were acquired in the axial plane using a 3D inversion recovery prepared fast spoiled gradient-echo sequence.^36^ Brain volumes were segmented into Automated Anatomical Labeling (AAL) ROIs using Statistical Parametric Mapping 12 (fil.ion.ucl.ac.uk/spm). Regional grey matter volumes were normalized for intracranial volume (ICV; (volume/ICVx1000).

We used publicly available MRI data for analyses. In ROS/MAP/MARS, cortical thickness measures were available. We therefore focused on a composite measure of cortical thickness in AD vulnerable regions, defined as the average thickness of bilateral entorhinal, parahippocampal, fusiform, inferior and middle temporal regions.^49,50^ In WRAP, cortical thickness data were not available, so we focused on a composite of volumetric MRI measures in equivalent regions. We examined longitudinal cortical thinning in ROS/MAP/MARS and cross-sectional brain volume in WRAP because publicly available follow-up data were limited in the latter dataset.

### 2.6. Plasma p-tau217

In WRAP, longitudinal plasma samples were analyzed for phosphorylated tau 217 (p-tau217) at the Michael T. Zuendel Biomarker Laboratory at Banner Sun Health Research Institute using the Roche cobas e801 platform. P-tau217 concentrations were quantified in pg/mL, and log-transformed prior to analyses.

### 2.7. Aβ Positron Emission Tomography (PET)

In WRAP, a subset of participants underwent PET imaging with either ^11^C-PiB or ^18^F-NAV4694 PET to quantify Aβ burden.^51^ Standardized uptake value ratios (SUVRs) were calculated for ROIs defined using the AAL atlas, with cerebellar gray matter as the reference region, as previously described.^52,53^ We examined a global cortical Aβ composite comprising the angular gyrus, anterior and posterior cingulate gyri, frontal medial orbital gyrus, precuneus, supramarginal gyrus, and middle and superior temporal gyri.^54^ Global Aβ-PET SUVRs were converted to centiloids to harmonize values across tracers.^55^ To characterize the sample, Aβ-PET positivity was defined using the previously established WRAP cutoff of 17.1 centiloids.^56^ PET analyses were performed cross-sectionally due to limited available follow-up in our analytic sample.

### 2.8. Analyses

Analysis of variance and χ^2^ tests summarized differences in participant characteristics by tertile of age at menopause. All models were adjusted for age at baseline, race, history of hormone therapy, and cause of menopause (spontaneous vs. surgical), as well as interactions with time for longitudinal models.

For episodic memory decline, linear mixed effects models in each dataset tested the three-way interaction between *APOE-ε4* status, age at menopause, and time on episodic memory scores, adjusting for the above covariates as well as years of education and its interaction with time. We included a spline term for baseline age to account for non-linear effects of aging. Random intercepts and slopes were included at the participant level, and study-level random intercepts were included in ROS/MAP/MARS.

For MRI brain atrophy, in ROS/MAP/MARS, linear mixed effects models tested the three-way interaction between *APOE-ε4*, age at menopause, and time on the composite measure. These models included random slopes and intercepts, as well a random intercept for MRI site. In WRAP, a linear model tested the two-way interaction between *APOE-ε4* and age at menopause on the composite measure.

For *in vivo* AD biomarkers in WRAP, a linear mixed effects model tested the three-way interaction between *APOE-ε4* status, age at menopause, and time on p-tau217, including random slopes and intercepts. Because Aβ-PET centiloid values were skewed, we used a robust linear regression model to test the interaction between *APOE-ε4* status and age at menopause on Aβ burden, adjusting for covariates described above and the PET tracer.

For all main models, we performed follow-up analyses stratified by age at menopause to estimate the magnitude of the effect of *APOE-ε4* with AD outcomes among women with earlier, average, and later tertiles of age at menopause. To contextualize findings in women, we also evaluated associations of *APOE-ε4* with AD outcomes in men using the same modelling approaches. Analyses were restricted to men aged ≥50 at baseline, to approximate the age range of postmenopausal women.

Although some studies suggest that *APOE-ε4* effects may differ by race or genetic ancestry,^9,10,57^ the sex-specific associations of *APOE ε4* with AD outcomes appear largely consistent across both White and Black individuals,^10–12^ which represent the two largest racial groups in our samples. As such, primary analyses included participants of all racial groups, with adjustment for race. In post hoc analyses, models were repeated separately in White and Black participants. Stratified analyses among Black participants in WRAP were not performed for brain volume or Aβ-PET due to small sample sizes.

Post hoc analyses also evaluated whether there were dose-dependent effects of *APOE-ε4* by testing the three-way interactions of *APOE ε4* allele dose (i.e., 2 copies or 1 copy vs. no copies), age at menopause, and time on AD outcomes. Given that body mass index (BMI) and smoking have been previously associated with both age at menopause and AD risk,^58–60^ sensitivity analyses repeated main models additionally adjusting for baseline BMI and smoking history, plus their interactions with time. We further examined the effects of *APOE-ε4* (i.e., *ε3/ε4* or *ε4/ε4*) and *APOE-ε2* (i.e., *ε2/ε3* or *ε2/ε2*) carriage relative to *ε3/ε3* homozygotes as the reference group.

## 3. Results

### Participant characteristics.

Demographic and clinical characteristics for participants in both datasets are presented in Table 1.

### Earlier menopause amplifies associations of APOE-ε4 with memory decline.

All main results are presented in Table 2. Cognitive analyses included 2,625 participants in ROS/MAP/MARS, and 495 participants in WRAP. In both datasets, there were significant three-way interactions between age at menopause, *APOE-ε4*, and time on episodic memory scores, such that *APOE-ε4* was more strongly related to memory decline in women with earlier (vs. later) menopause.

### Earlier menopause exacerbates associations of APOE-ε4 with brain atrophy.

Longitudinal MRI analyses included 533 participants in ROS/MAP/MARS. There was a significant three-way interaction between *APOE-ε4*, age at menopause, and time on cortical thinning, indicating that *APOE-ε4* was more strongly linked to greater atrophy in women with earlier (vs. later) menopause. In WRAP (*N*=173), there was also a significant interaction between *APOE-ε4* and age at menopause on cross-sectional brain volume, such that earlier menopause strengthened the association between *APOE-ε4* and smaller volume.

### Earlier menopause strengthens the links between APOE-ε4 and AD biomarkers.

Plasma analyses included 495 WRAP participants. There was a three-way interaction between age at menopause, *APOE-ε4*, and time on plasma p-tau217, such that earlier menopause exacerbated the associations between *APOE-ε4* and longitudinal p-tau217 accumulation. Aβ-PET analyses included 183 WRAP participants (*N*=175 ^11^C-PiB and *N*=8 ^18^F-NAV4694). *APOE-ε4* interacted with age at menopause such that earlier menopause strengthened the association between *APOE-ε4* and greater Aβ burden.

### Stepwise increases in APOE-ε4 effect magnitude with earlier ages at menopause.

Across both datasets and outcomes, analyses stratified by age at menopause (tertiles) showed that *APOE-ε4* associations were stronger in women with earlier (vs. later) ages at menopause.

### Age at menopause may modulate sex differences in APOE-ε4 associated risk

To contextualize findings observed in women, post hoc analyses evaluated associations of *APOE-ε4* and AD outcomes in men (demographics and sample sizes in eTable 1) and compared their magnitudes with corresponding associations in women across tertiles of age at menopause (Table 2). Across most outcomes, the effect sizes for *APOE-ε4* in women with earlier menopause were larger than those observed in men, whereas *APOE-ε4* effect sizes in women with average or later menopause were more similar to those in men. These findings suggest that the sex differences in *APOE-ε4* associated risk may partially depend on age at menopause in women.

### Post hoc analyses

We re-estimated models stratified by race in each dataset. Demographic characteristics of White and Black participants are presented in eTable 2. Generally, the magnitudes of the interactive associations of *APOE-ε4* and age at menopause on AD outcomes appeared larger in Black/African American participants relative to White participants (eTable 3). Post hoc analyses examining *APOE-ε4* dose effects showed stronger associations between *APOE-ε4* and age at menopause among *ε4* homozygotes for memory decline in ROS/MAP/MARS and p-tau217 accumulation in WRAP, but not for other outcomes (eTable 4).

### Sensitivity analyses

Re-estimating the primary models with additional adjustment for baseline BMI and smoking history yielded consistent findings (eTable 5). Additional analyses comparing *APOE-ε4* and *APOE-ε2* carriers with *ε3/ε3* homozygotes showed similar results for *APOE-ε4 and* no evidence of an interaction between age at menopause and *APOE-ε2* (eTable 6).

## 4. Discussion

Across two independent datasets, age at menopause modified the associations between *APOE-ε4* and multiple AD-related outcomes. Specifically, *APOE-ε4* was associated with greater memory decline, brain atrophy, and *in vivo* markers of AD pathology in women with earlier (vs. later) menopause. These convergent effects on AD endophenotypes support the robustness of the observed interaction and are consistent with preclinical and emerging human evidence suggesting that menopause-related endocrine changes may exacerbate *APOE-ε4* associated AD risk.^15,29–31^ When compared with men, women with earlier menopause exhibited larger *APOE-ε4* associated effects, whereas women with later menopause generally showed effect sizes more similar to those observed in men. Together, these findings highlight menopause timing as an important modifier of *APOE-ε4* contributions to AD risk and underscore the importance of endocrine processes in influencing vulnerability to dementia.

The mechanisms linking earlier menopause to increased *APOE-ε4* associated AD risk remain unclear. *APOE-ε4* promotes AD risk through multiple pathways, including Aβ aggregation and impaired clearance, increased tau pathology, metabolic and cholesterol transport dysregulation, synaptic plasticity changes, blood–brain barrier and cerebrovascular dysfunction, and enhanced neuroinflammation.^61–63^ Estradiol influences many of these same processes and may help mitigate some of the adverse effects of *APOE-ε4* carriage.^64–66^ Consequently, earlier loss of estradiol-mediated neuroprotection (alongside earlier accumulation of potentially neurotoxic effects of FSH^15,67^) may amplify *APOE-ε4* related susceptibility to synaptic, vascular, and inflammatory pathways involved in AD pathogenesis.^15,16,29^ Consistent with this hypothesis, previous research has reported that women with earlier menopause may be at heightened risk for synaptic and vascular contributions to AD,^22,23^ effects which may be especially salient in *APOE-ε4* carriers.

Compared to men, women with earlier menopause exhibited larger *APOE-ε4* associated effects, whereas women with later menopause generally showed effect sizes more similar to those observed in men. These findings suggest that menopause timing may contribute to previously observed sex differences in *APOE-ε4* associated neurodegenerative risk,^6–12^ highlighting the importance of considering within-sex heterogeneity in understanding sex-specific dementia risk.^68^ Post hoc analyses suggested that the interactive associations of *APOE-ε4* and age at menopause may be more pronounced in Black/African American (vs. White) participants. However, these findings should be interpreted cautiously given limited statistical power to formally assess racial/ethnic differences, particularly in WRAP. These observations underscore the urgent need for greater diversity in aging research.

This study has several strengths. First, across two large datasets we found that earlier age at menopause amplifies the effects of *APOE-ε4* on multiple AD outcomes. Notably, these findings emerged despite substantial differences between datasets in recruitment strategies (community-dwelling and relatively healthy in ROS/MAP/MARS vs. mostly adult children of patients with AD in WRAP) and demographics (ROS/MAP/MARS was substantially older than WRAP).

There were also limitations. Menopause history was self-reported and may therefore be subject to recall error, and reproductive health history questionnaires in both datasets were limited in detail. Future studies including comprehensive and prospectively collected data on women’s reproductive health history could more accurately ascertain age at menopause. Additionally, participants in both datasets were well-educated, and included limited representation of racial/ethnic groups beyond White or Black/African American. Further research in diverse global samples is needed to better understand how female-specific *APOE-ε4* effects may influence AD risk among individuals of different racial/ethnic, socioeconomic, and cultural backgrounds.

## 5. Conclusion

Age at menopause modified the association of *APOE-ε4* with multiple AD outcomes across two independent datasets, such that earlier menopause amplified the detrimental influence of *APOE-ε4* on memory decline, brain atrophy, and *in vivo* markers of AD pathology. These findings suggest that menopause timing may moderate susceptibility to AD especially among *APOE-ε4* carriers and identify reproductive aging as a potential pathway linking well-established sex differences in genetic risk to neurodegeneration in women. Incorporating menopause history into studies of cognitive and brain aging is important for advancing our understanding of AD risk.

## Supplementary Material

Supplementary Files

This is a list of supplementary files associated with this preprint. Click to download.

- menoAPOE4supplement.pdf

## Figures and Tables

**Figure 1.**
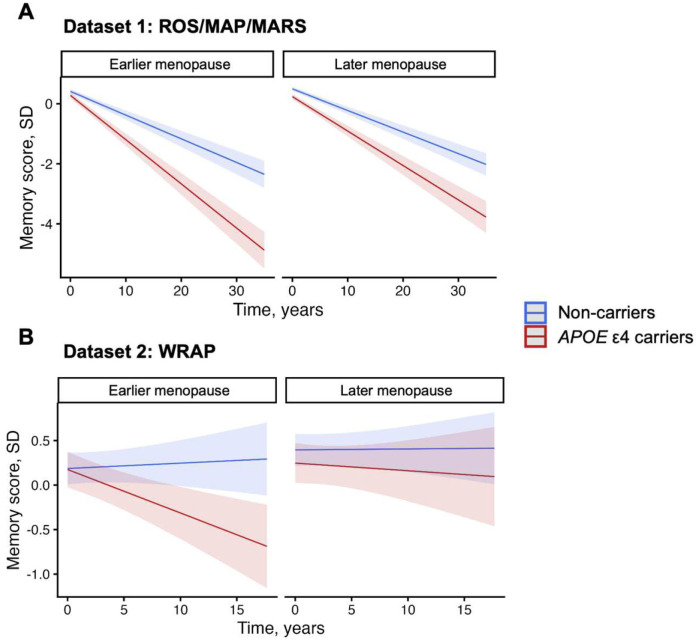
Earlier menopause exacerbates the associations of *APOE-ε4* with episodic memory decline. The plots depict the three-way interactions between *APOE-*ε4 carriage, age at menopause, and time on episodic memory scores in both datasets, adjusted for age, years of education, cause of menopause (spontaneous vs. surgical), history of hormone therapy, race, and their interactions with time, in (**A**) Dataset 1: ROS/MAP/MARS and (**B**) Dataset 2: WRAP. Age at menopause was modelled continuously and is shown at 20^th^ (i.e., earlier) and 80^th^ (i.e., later) percentiles for visualization purposes. Shaded regions are 95% confidence intervals.

**Figure 2.**
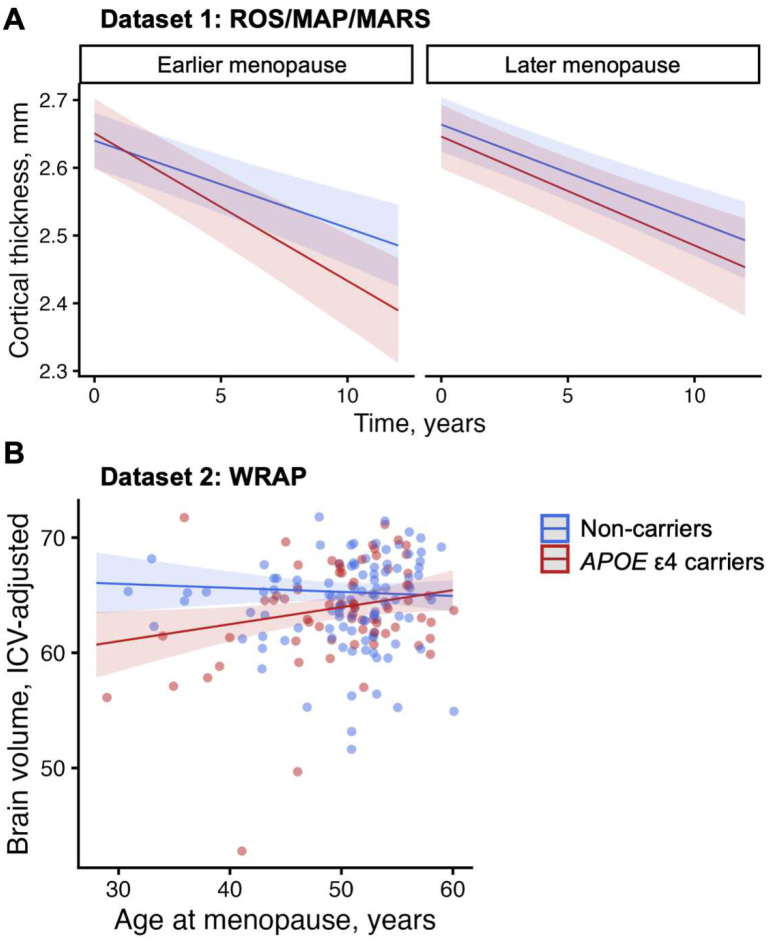
Earlier menopause strengthens the associations of *APOE*-ε4 with brain atrophy in AD vulnerable regions. (**A**): The three-way interaction between *APOE*-ε4 carriage, age at menopause, and time on cortical thickness in AD vulnerable regions (in mm) in Dataset 1: ROS/MAP/MARS, adjusted for baseline age, cause of menopause (spontaneous vs. surgical), history of hormone therapy, race, and their interactions with time. Age at menopause was modelled continuously and is shown at 20^th^ (i.e., earlier) and 80^th^ (i.e., later) percentiles for visualization purposes. (**B**): The two-way interaction between *APOE*-ε4 carriage and age at menopause on brain volume in AD vulnerable regions (adjusted for intracranial volume) in Dataset 2: WRAP. The model is adjusted for age, cause of menopause (spontaneous vs. surgical), history of hormone therapy, and race. In both panels, shaded regions represent 95% confidence intervals.

**Figure 3.**
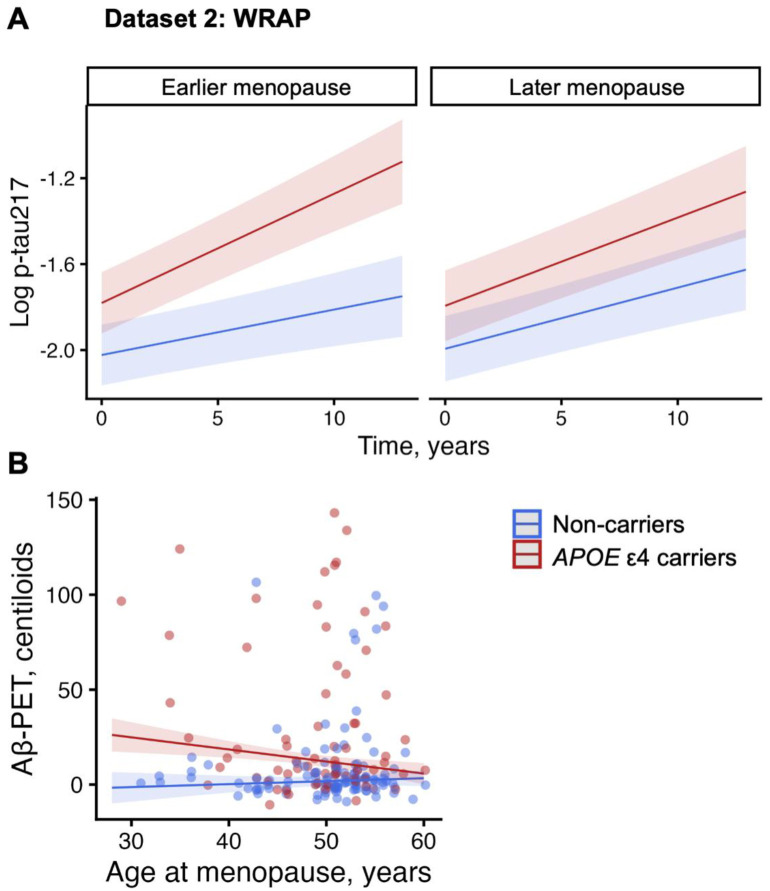
Earlier menopause exacerbates the associations of *APOE*-ε4 with AD pathology. (**A**) The three-way interaction between *APOE*-ε4 carriage, age at menopause, and time on plasma p-tau217 (log-transformed) in Dataset 2: WRAP, adjusted for age, cause of menopause (spontaneous vs. surgical), history of hormone therapy, their interactions with time, and race. Age at menopause was modelled continuously and is shown at 20^th^ (i.e., earlier) and 80^th^ (i.e., later) percentiles for visualization purposes. (**B**) The two-way interaction between *APOE-ε4* carriage and age at menopause global cortical Aβ-PET in centiloids in Dataset 2: WRAP, adjusted for age, cause of menopause (spontaneous vs. surgical), history of hormone therapy, race, and PET tracer. In both panels, shaded regions represent 95% confidence intervals.

**Table 1.** Participant characteristics.

	Dataset 1: ROS/MAP/MARS					Dataset 2: WRAP				
	All women *N* = 2,625	Age at menopause tertile				All women, *N* = 512	Age at menopause tertile			
		Earlier (<45) *N* = 787	Average (≥45 or ≤51) *N* = 1,104	Later (>51) *N* = 734	P value		Earlier (<50) *N* = 165	Average (≥50 or ≤53) *N* = 204	Later (>53) *N* = 143	P value
Age at baseline, mean (SD)	77.4 (7.77)	76.7 (7.62)	77.9 (7.80)	77.5 (7.85)	.005	60.2 (5.57)	60.5 (6.33)	59.5 (5.49)	60.8 (4.59)	.07
Years of education, mean (SD)	15.7 (3.57)	15.4 (3.43)	16.1 (3.70)	15.6 (3.47)	<.001	16.0 (2.77)	15.8 (2.73)	16.0 (2.69)	16.2 (2.95)	.49
Study, *N* (%)										
ROS	878 (33.4)	259 (32.9)	394 (35.7)	225 (30.7)						
MAP	1,252 (47.7)	315 (40.0)	558 (50.5)	379 (51.6)						
MARS	495 (18.9)	213 (27.1)	152 (13.8)	130 (17.7)	<.001	-	-	-	-	-
Race, *N* (%)										
White	1,977 (75.1)	508 (64.5)	891(80.7)	573 (78.1)		456 (89.1)	136 (82.4)	187 (91.7)	133 (93.0)	
Black or African American	633 (24.1)	273 (34.7)	203 (18.4)	157 (21.4)		50 (9.8)	28 (17.0)	14 (6.9)	8 (5.6)	
American Indian or Alaska Native	9 (0.3)	3 (0.4)	4 (0.4)	2 (0.3)		0 (0.0)	0 (0.0)	0 (0.0)	0 (0.0)	
Native Hawaiian or Other Pacific Islander	1 (0.0)	0 (0.0)	0 (0.0)	1 (0.1)		0 (0.0)	0 (0.0)	0 (0.0)	0 (0.0)	
Asian	6 (0.2)	2 (0.3)	3 (0.3)	1 (0.1)		0 (0.0)	0 (0.0)	0 (0.0)	0 (0.0)	
Unknown	4 (0.2)	1 (0.1)	3 (0.3)	0 (0.0)	<.001	6 (1.2)	1 (0.6)	3 (1.5)	2 (1.4)	.005
*APOE, N* (%)										
ε2/ε2	16 (0.6)	7 (0.9)	5 (0.5)	4 (0.5)		4 (0.8)	1 (0.6)	0 (0.0)	3 (2.1)	
ε2/ε3	339 (12.9)	95 (12.1)	165 (14.9)	79 (10.8)		38 (7.4)	15 (9.1)	13 (6.4)	10 (7.0)	
ε3/ε3	1,616 (61.6)	473 (60.1)	671 (60.8)	472 (64.3)		265 (51.8)	80 (48.5)	113 (55.4)	72 (50.3)	
ε3/ε4	588 (22.4)	185 (23.5)	237 (21.5)	166 (22.6)		180 (35.2)	60 (36.4)	68 (33.3)	52 (36.4)	
ε4/ε4	66 (2.5)	27 (3.4)	26 (2.4)	13 (1.8)	0.07	25 (4.9)	9 (5.5)	10 (4.9)	6 (4.2)	.51
Age at menopause, mean (SD)	47.5 (7.32)	38.5 (4.78)	48.8 (2.15)	55.3 (3.05)	<.001	50.1 (6.29)	43.1 (6.17)	51.6 (1.05)	55.9 (1.80)	<.001
Surgical menopause, *N* (%)	956 (36.4)	544 (69.1)	302 (27.4)	110 (15.0)	<.001	15 (2.9)	8 (4.8)	3 (1.5)	4 (2.8)	.16
History of hormone therapy, *N* (%)	958 (36.5)	296 (37.6)	415 (37.6)	247 (33.7)	.16	256 (50.0)	102 (61.8)	100 (49.0)	54 (37.8)	<.001
Cognitive diagnosis at study entry (%)										
No cognitive impairment	1,926 (73.4)	556 (70.6)	821 (74.4)	549 (74.8)		502 (98.0)	161 (97.6)	200 (98.0)	141 (98.6)	
MCI	607 (23.2)	208 (26.4)	242 (22.0)	156 (21.2)		10 (2.0)	4 (2.4)	4 (2.0)	2 (1.4)	
Dementia	92 (3.5)	23 (2.9)	40 (3.7)	29 (4.0)	0.10	0 (0.0)	0 (0.0)	0 (0.0)	0 (0.0)	.30
BMI, mean (SD)	27.9 (5.86)	28.7 (6.09)	27.4 (5.55)	27.9 (6.00)	<.001	29.2 (6.98)	29.6 (7.63)	28.7 (6.74)	29.4 (6.54)	.42
Ever smoked, *N* (%)	791 (30.2)	261 (33.2)	315 (28.5)	215 (29.3)	.08	210 (41.3)	70 (42.4)	90 (44.3)	50 (35.5)	.24
Total follow-up, mean (SD) years	9.25 (6.18)	9.09 (6.07)	9.01 (6.24)	9.78 (6.19)	.03	5.28 (3.35)	4.03 (1.68)	5.32 (3.07)	6.67 (4.48)	<.001
Number of longitudinal cognitive visits, median (IQR)	8 (5, 12)	8 (4, 12)	8 (5, 12)	9 (6, 13)	.03	3 (2, 5)	3 (2, 5)	3.5 (2, 5)	3 (2, 5)	.62
Number of longitudinal MRI visits, median (IQR)	2 (1, 4)	2 (1, 4)	2 (1, 4)	2 (1, 4)	.30	-	-	-	-	-
Number of longitudinal plasma visits, median (IQR)	-	-	-	-	-	3 (2, 4)	3 (2, 4)	3 (2, 4)	3 (2, 4)	.35
Aβ-PET positivity, *N* (%)	-	-	-	-	-	47 (24.0)	15 (23.8)	21 (26.3)	11 (20.8)	.77

**Table 2.** Results.

Model	Interaction of *APOE-ε4* and age at menopause, all participants		Main association of *APOE-ε4*, stratified by age at menopause						Main association of *APOE-ε4* in men	
			*Earlier menopause*		*Average menopause*		*Later menopause*			
	β (95% CI)	*p*	β (95% CI)	*p*	β (95% CI)	*p*	β (95% CI)	*p*	β (95% CI)	*p*
**Dataset 1: ROS/MAP/MARS**										
Longitudinal memory	0.068 (0.012, 0.124)	.02	−0.418 (−0.523, −0.312)	<.001	−0.262 (−0.357, −0.167)	<.001	−0.181 (−0.290, −0.073)	.001	−0.358 (−0.455, −0.262)	<.001
Longitudinal cortical thickness	0.071 (0.001, 0.142)	.05	−0.131 (−0.265, 0.004)	.06	−0.109 (−0.212, −0.006)	.04	0.017 (−0.125, 0.158)	.82	−0.161 (−0.365, 0.044)	.12
**Dataset 2: WRAP**										
Longitudinal memory	0.092 (0.010, 0.175)	.03	−0.198 (−0.366, −0.031)	.02	−0.118 (−0.233, −0.002)	.05	−0.113 (−0.293, 0.066)	.22	−0.028 (−0.098, 0.042)	.43
Cross-sectional brain volume	0.256 (0.019, 0.494)	.03	−0.622 (−1.008, −0.235)	.002	0.107 (−0.311, 0.525)	.61	−0.530 (−1.133, 0.072)	.08	−0.029 (−0.367, 0.310)	.87
Longitudinal p-tau217	−0.067 (−0.012, 0.001)	.05	0.192 (0.057, 0.328)	.005	0.150 (0.058, 0.242)	.001	0.101 (−0.004, 0.215)	.06	0.123 (0.051, 0.200)	.001
Cross-sectional Aβ-PET	−0.146 (−0.262, −0.031)	.01	0.367 (0.176, 0.558)	<.001	0.392 (0.182, 0.602)	<.001	0.239 (−0.019, 0.500)	.07	0.308 (0.151, 0.465)	<.001

## Key Points

### Question:

Does age at menopause modify associations between *APOE-ε4* and Alzheimer’s disease (AD) outcomes?

### Findings:

In two independent datasets of postmenopausal women, earlier age at menopause strengthened associations of *APOE-ε4* with memory decline and brain atrophy. In a dataset with *in vivo* biomarkers of AD pathology, earlier menopause also amplified associations of *APOE-ε4* with plasma p-tau217 accumulation and cortical Aβ-PET burden.

### Meaning:

Earlier menopause may increase susceptibility to *APOE*-ε4–related AD processes, highlighting midlife endocrine processes as potential targets for interventions to mitigate genetic risk for AD in women.
